# Acute Quadriparesis: A Rare Presenting Manifestation of an Adrenal Tumor

**DOI:** 10.7759/cureus.68395

**Published:** 2024-09-01

**Authors:** Subbiah Senthilnathan, Keesari Sai Sandeep Reddy, Chakradhar Ravipati

**Affiliations:** 1 Internal Medicine, Saveetha Medical College and Hospital, Saveetha Institute of Medical and Technical Sciences, Saveetha University, Chennai, IND; 2 Radiodiagnosis, Saveetha Medical College and Hospital, Saveetha Institute of Medical and Technical Sciences, Saveetha University, Chennai, IND

**Keywords:** endocrine disorders, elevated serum aldosterone, hypokalemia, adrenal tumor, hypokalemic quadriparesis

## Abstract

Acute quadriparesis is caused by severe and sudden weakness of all four limbs, which is a distressing clinical presentation that demands immediate and comprehensive investigation. This case report presents a unique instance of acute quadriparesis secondary to an adrenal tumor. A 54-year-old female presented with acute weakness in her upper and lower limbs over six hours without a prior history of fever, convulsions, or other systemic symptoms. Laboratory evaluations revealed significant hypokalemia, prompting further investigation. Differential diagnoses such as Guillain-Barré syndrome, demyelinating lesions, and myopathy were systematically ruled out through clinical evaluation and diagnostic testing. The patient's hypokalemia was aggressively managed with intravenous potassium replacement, leading to significant improvement in muscle strength. Radiological imaging revealed a hyperenhancing lesion in the left adrenal gland, consistent with an adrenal tumor. Elevated serum aldosterone levels supported the diagnosis of hyperaldosteronism. The patient's condition stabilized with intravenous potassium and antihypertensive medications, and a laparoscopic adrenalectomy was performed to remove the adrenal tumor. Postoperatively, the patient's blood pressure and electrolyte levels normalized, and she experienced a full recovery of muscle strength. This case highlights the importance of considering endocrine disorders in the differential diagnosis of acute quadriparesis and underscores the need for a comprehensive diagnostic approach, including routine electrolyte assessments, hormonal evaluations, and thorough imaging studies. Effective management involving prompt identification and treatment of underlying causes is critical for optimal patient outcomes. This case contributes valuable insights into the diverse clinical manifestations of adrenal tumors and the importance of early and accurate diagnosis.

## Introduction

Acute quadriparesis, characterized by sudden and severe weakness in all four limbs, is an alarming clinical presentation that requires prompt and thorough investigation to identify the underlying cause. While it is commonly associated with neurological conditions such as Guillain-Barré syndrome, spinal cord injuries, and metabolic disturbances, it is rarely seen as the initial manifestation of an adrenal tumor. This case report presents a unique instance of acute quadriparesis secondary to an adrenal tumor, underscoring the importance of considering a broad differential diagnosis in such presentations. The aim is to provide insights into this rare condition's clinical features, diagnostic challenges, and management strategies. Adrenal tumors are relatively uncommon, with an estimated prevalence of 4%-7% in the general population, often detected incidentally during imaging studies performed for other reasons. These tumors can be classified as benign (adenomas) or malignant (adrenocortical carcinoma, pheochromocytoma), with varying clinical presentations depending on their size, location, and hormonal activity [1,2].

Functional adrenal tumors, such as pheochromocytomas, secrete excessive catecholamines, leading to a range of symptoms, including hypertension, palpitations, and hyperglycemia. However, they can also present with less typical symptoms, such as neurological deficits, which are frequently not immediately attributed to an adrenal etiology [3]. This case involves a patient presenting with acute quadriparesis, which led to the discovery of an adrenal tumor during the diagnostic workup. The clinical course, diagnostic challenges, and treatment outcomes are detailed to highlight this rare presentation's significance and contribute to the existing body of knowledge on adrenal tumors.

## Case presentation

A 54-year-old female patient who had no known comorbidities arrived at the emergency room with an acute case of weakness in her upper and lower limbs. Over six hours, she was unable to lift her upper limbs, wake up from bed, or stand and walk. There was no prior history of fever, convulsions, or visual blurriness. Hearing impairment, no complaints of (C/O) ear discharge. No coughing up expectoration of sputum and dyspnea. No loose stools, bleeding diathesis, or burning micturition. There was no noteworthy history of such incidents, and she had C/O loss of appetite over the previous three months.

Upon assessment, the patient was found to be afebrile, conscious, and aware of time, place, and people. At admission, her vital signs were stable, with capillary blood glucose (CBG) measuring 240 mg/dL and Glasgow Coma Scale (GCS) at E4V5M4 (eye, verbal, motor). Normal higher mental function was found throughout the neurological examination. There is no papilledema, neck stiffness, or abnormal findings suggestive of cranial nerve palsy. Examination revealed flaccid weakness, areflexia of bilateral upper and lower extremities, and decreased muscle strength of 3/5 in the lower and 4/5 in the upper extremities. Plantar reflex was mute on both sides. The sensation of touch, pain, and proprioception was preserved.

On the second day of admission, the patient remained tachypneic and maintained a room air peripheral capillary oxygen saturation (SpO_2_) of 78%-80% due to possible acute respiratory distress syndrome (ARDS). Arterial blood gas (ABG) measurements in room air revealed a pH of 7.579, partial pressure of carbon dioxide (pCO_2_) of 33.7, partial pressure of oxygen (pO_2_) of 39 mmHg, bicarbonate (HCO_3_) of 31.6, and SpO_2_ of 95%. Interpretation: Based on Berlin's criteria, the diagnosis of moderate ARDS was made with a partial pressure of oxygen/fraction of inspired oxygen (PaO_2_/FiO_2_) of 114%. The patient was fitted with a mechanical ventilator and electively intubated. The patient was experimentally put on 500 mg of tab Azee and 4.5 g Tds of injection PipTaz. The patient was extubated after breathing independently on day nine of admission and tolerating 3 L of oxygen on a T-piece. Following extubating, the patient maintained a 95% SpO_2_ in room air. The blood and urine investigations are presented in Table 1, which are suggestive of severe hypokalemia, renal potassium wasting, transaminitis, and poorly controlled diabetes.

**Table 1:** Complete blood count, serum electrolytes, osmolality, renal function test, liver function test, glycated hemoglobin, electrolytes, and thyroid profile

Investigations	Results	Reference Values	Interpretation
Complete blood count	Hb: 11.6 g/dL	Males: 13.8–17.2 g/dL	Within normal limits, not suggestive of chronic iron or vitamin B12 deficiency
		Females: 12.1–15.1 g/dL	
	RBC: 5.48 million/cu.mm	Males: 4.7–6.1 million/cu.mm	
		Females: 4.2–5.4 million/cu.mm	
	Total leucocyte count: 9200 cells/cu.mm	4,000–11,000 cells/cu.mm	
	Platelets: 2 lacs/cu.mm	150,000–450,000/cu.mm	
	Vitamin B12: 712 pg/mL	200–900 pg/mL	
Serum electrolytes	Sodium: 143 meq/dL	135–145 mEq/L	Suggestive of severe hypokalemia
	Potassium: 1.2 meq/dL	3.5–5.0 mEq/L	
	Chloride: 85 meq/dL	98–106 mEq/L	
	Bicarbonate: 40 meq/dL	22–28 mEq/L	
Osmolality	Serum osmolality: 282 mosm/kg	275–295 mosm/kg	Suggestive of renal potassium wasting
	Urine osmolality: 438 mosm/kg	50–1200 mosm/kg	
	Urine spot potassium: 43.5 meq/L	25–125 mEq/L	
	Transtubular potassium gradient: 13.4	Typically 8–9 in normal conditions	
Renal function test	Serum urea: 44 mg/dL	7–20 mg/dL	Within normal limits
	Serum creatinine: 0.7 mg/dL	Males: 0.74–1.35 mg/dL	
		Females: 0.59–1.04 mg/dL	
Liver function test	Aspartate transaminase: 143 u/L	10–40 U/L	Suggestive of transaminitis
	Alanine transaminase: 141 u/L	7–56 U/L	
	Alkaline phosphatase: 189 u/L	44–147 U/L	
Glycated hemoglobin	HbA1c: 7.4 g/dL	Normal: below 5.7%	Suggestive of poorly controlled diabetes
		Prediabetes: 5.7%–6.4%	
		Diabetes: 6.5% or higher	
Electrolytes	Corrected calcium: 8.4 mg/dL	8.5–10.5 mg/dL	Not suggestive of mineral and bone disorder
	Serum phosphorous: 2.8 mg/dL	2.5–4.5 mg/dL	
	Serum uric acid: 2.5 mg/dL	Males: 3.4–7.0 mg/dL	
		Females: 2.4–6.0 mg/dL	
	Vitamin D: 46 ng/mL	20–50 ng/mL	
Thyroid profile	Free T3: 2.85 pg/mL	2.0–4.4 pg/mL	Not suggestive of thyrotoxicosis
	Free T4: 0.95 ng/dL	0.8–2.0 ng/dL	
	TSH: 2.25 mIU/L	0.5–4.5 mIU/L	

A routine investigation reveals significant hypokalemia, which may be the cause of the four limbs' extreme paralysis. Nevertheless, it is crucial to rule out alternative etiologies. After receiving intravenous potassium replacement therapy, the patient's muscle power recovered almost entirely by the third day. A transtubular potassium gradient of 13.4 indicates significant renal potassium loss, most likely brought on by hyperaldosteronism-causing factors.

**Figure 1:**
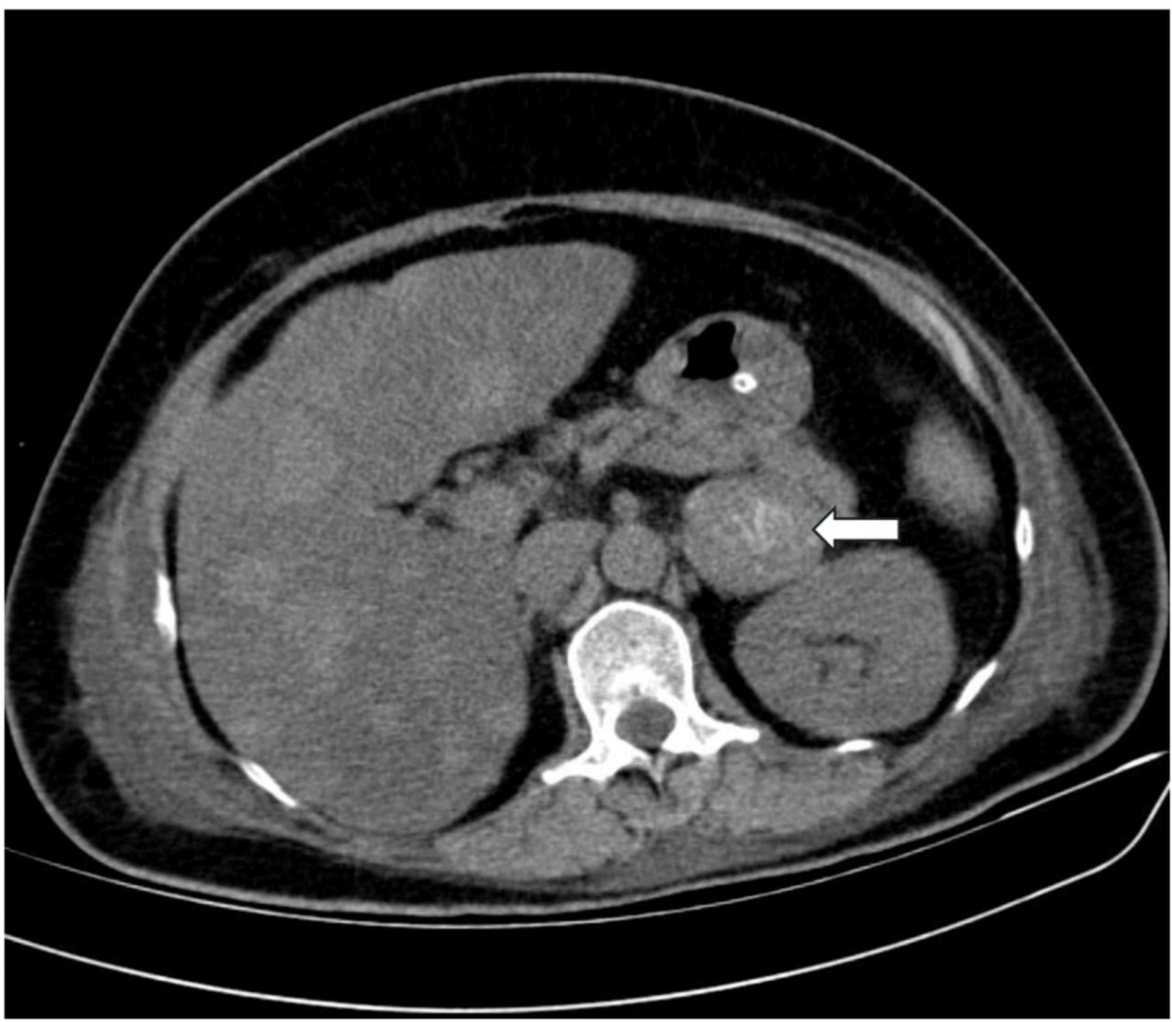
CT abdomen axial section showing a left suprarenal mass (white arrow)

**Figure 2:**
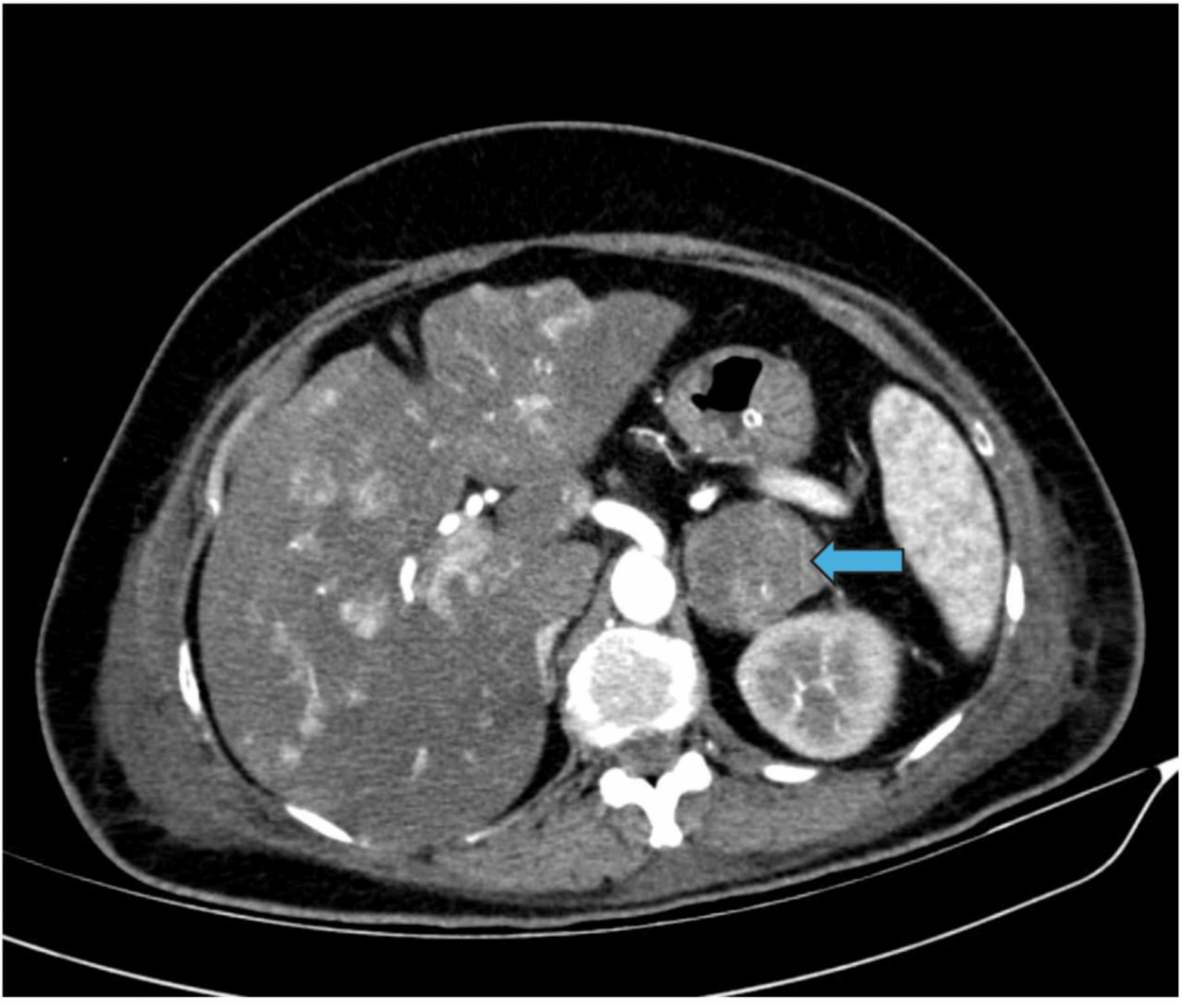
Contrast-enhanced CT abdomen showing a heterogenous enhancing mass in the left suprarenal region (blue arrow)

A contrast-enhanced computed tomography (CECT) of the abdomen was performed due to suspicions of an adrenal tumor, and the results showed a single, heterogeneously hyperenhancing lesion in the left adrenal gland (Figure 1) that measures 4.1 × 4.2 × 6.2 cm, with the absence of macroscopic fat stranding evidence. There is no notable regional lymphadenopathy. Heterogeneous hyperenhancement is primarily seen in the venous phase post-contrast (Figure 2). Subsequently, the patient underwent investigation for refractory hypokalemia with secondary hypertension, as shown in Table 2.

**Table 2:** Investigation done for refractory hypokalemia with secondary hypertension

Parameter	Value	Reference Range
8 a.m. cortisol	8.12	6.2- 19.4
Serum aldosterone	540 pg/mL	25-315 pg/mL
Serum renin	2.32 ng/mL	1.5- 8 ng/mL
Plasma metanephrine	48 pg/mL	<65 pg/mL
Plasma normetanephrine	82 pg/mL	<196 pg/mL
Urinary vanillylmandelic acid	4.5 mg/g	2-7 mg/g

She was started on multiple classes of antihypertensive medications, such as angiotensin receptor blockers (ARB), calcium channel blockers, and alpha-blockers. Her blood pressure was brought under control. The left suprarenal tumor was removed by laparoscopic adrenalectomy, and antihypertensives were administered both before and during the procedure. The procedure and the recovery phase were both uneventful.

## Discussion

In this case of acute quadriparesis with an adrenal tumor, the patient's hypokalemia was aggressively managed with intravenous potassium replacement, resulting in significant improvement in muscle strength by the third day of admission. The transtubular potassium gradient of 13.4 suggested substantial renal potassium loss, likely due to hyperaldosteronism. Radiological imaging with a CECT scan of the abdomen revealed a hyperenhancing lesion in the left adrenal gland, consistent with an adrenal tumor. Elevated serum aldosterone levels further supported the diagnosis of hyperaldosteronism. The patient's condition was stabilized with a combination of intravenous potassium and antihypertensive medications, including alpha-blockers, calcium channel blockers, and ARB. Following stabilization, a laparoscopic adrenalectomy was performed to remove the adrenal tumor. Postoperatively, the patient's blood pressure and electrolyte levels normalized, and she experienced a full recovery of muscle strength.

Kamijo et al. (1998) [4] reported a case of a 19-year-old woman who developed quadriparesis after inhaling lacquer thinner containing toluene. Despite potassium replacement therapy, her condition worsened, leading to adrenal insufficiency secondary to bilateral adrenal hemorrhage, resulting in death. This case highlights the potential for adrenal involvement in acute quadriparesis and the critical importance of early recognition and management. Modi et al. (2020) [5] described a 46-year-old male initially diagnosed with an intradural spinal tumor at L1, presenting with paraparesis that progressed to quadriparesis. Hypokalemia was discovered, caused by primary hyperaldosteronism due to a right adrenal adenoma. Potassium supplementation and spironolactone treatment led to complete recovery within 72 hours. This case underscores the need to consider metabolic causes in the differential diagnosis of acute quadriparesis.

Rahmani et al. (2021) [6] presented a case of a 49-year-old man with a recurrent neuroendocrine tumor (NET) of adrenal origin. The patient initially presented with hypercortisolism and severe hypokalemia, leading to quadriparesis. Following bilateral adrenalectomy, the patient experienced a prolonged disease-free period but later developed a pulmonary carcinoid tumor with elevated adrenocorticotropic hormone (ACTH) levels, indicating a secondary focus of the NET. This case emphasizes the importance of long-term follow-up in patients with ACTH-secreting adrenal NETs. Fisher et al. (1987) [7] reported a 40-year-old woman presenting with watery diarrhea syndrome and acute quadriparesis. Biochemical testing revealed severe hypokalemia and elevated plasma levels of vasoactive intestinal polypeptide (VIP), leading to the diagnosis of a VIP-secreting pheochromocytoma. Surgical removal of the adrenal tumor resolved her symptoms and normalized hormone levels. This case illustrates the diverse clinical presentations of adrenal tumors and the significance of hormonal assessments.

Başarslan et al. (2014) [8] documented a case of a 45-year-old male who presented with acute quadriparesis and severe hypokalemia. The patient's muscle strength recovered completely with potassium supplementation, highlighting hypokalemia as a reversible cause of acute flaccid quadriparesis. This case reinforces the need for clinicians to consider and promptly correct electrolyte imbalances in similar presentations. Mandreker et al. (2018) [9] described a 42-year-old woman who developed acute-onset quadriparesis due to severe hypokalemia and hypernatremia. She was diagnosed with hypophysitis-induced panhypopituitarism, leading to neurogenic diabetes insipidus. Treatment with intravenous potassium, fluids, and hormone replacement resulted in complete recovery. This case highlights the rare endocrine causes of acute quadriparesis.

Garg et al. (2014) [10] reported a case of a 73-year-old female with rapidly progressive ascending paraparesis that progressed to quadriparesis due to hyperkalemia. Prompt antihyperkalemic treatment led to a dramatic improvement as potassium levels normalized. This case underscores the importance of considering hyperkalemia in the differential diagnosis of acute flaccid quadriparesis. Jhamb et al. (2007) [11] presented the case of a 22-year-old woman with acute-onset flaccid quadriparesis due to severe hypokalemia and metabolic acidosis. The underlying cause was identified as diffuse lymphomatous infiltration of the kidneys, leading to renal tubular acidosis. This case illustrates the potential for hematological malignancies to present with neurological symptoms.

Wahab et al. (2011) [12] documented a 56-year-old diabetic woman who presented with acute flaccid quadriparesis and hyperkalemia. The electrocardiogram showed a sine wave pattern, indicating severe hyperkalemia. The patient improved significantly after treatment with calcium gluconate, insulin, and dextrose. This case highlights the critical nature of hyperkalemia and the need for timely intervention.

## Conclusions

This case report highlights the significance of considering endocrine disorders, such as adrenal tumors, in the differential diagnosis of acute quadriparesis. The patient's presentation with severe muscle weakness in all four limbs due to profound hypokalemia secondary to hyperaldosteronism highlights the critical importance of a comprehensive diagnostic approach. This includes routine electrolyte assessments, hormonal evaluations, and thorough imaging studies. Effective management, involving intravenous potassium replacement and surgical removal of the adrenal tumor, resulted in a complete recovery of muscle strength. This case emphasizes the need for clinicians to maintain a broad differential diagnosis, promptly identify and treat underlying causes, and adopt an interdisciplinary approach to patient care. It contributes valuable insights into the diverse clinical manifestations of adrenal tumors and the importance of early and accurate diagnosis in achieving optimal patient outcomes.

## References

[1] He X, Peter PR, Auchus RJ (2021). Approach to the patient with an incidental adrenal mass. Med Clin North Am.

[2] Kikuchi Y, Wada R, Sakihara S, Suda T, Yagihashi S (2012). Pheochromocytoma with histologic transformation to composite type, complicated by watery diarrhea, hypokalemia, and achlorhydria syndrome. Endocr Pract.

[3] Lenders JW, Eisenhofer G, Mannelli M (2005). Phaeochromocytoma. Lancet.

[4] Kamijo Y, Soma K, Hasegawa I, Ohwada T (1998). Fatal bilateral adrenal hemorrhage following acute toluene poisoning: a case report. J Toxicol Clin Toxicol.

[5] Modi HN, Shreshtha U, Lakhani O (2020). Hypokalemic paraparesis progressing to quadriparesis in a case of intradural spinal tumor. J Orthop Case Rep.

[6] Rahmani F, Tohidi M, Dehghani M, Broumand B, Hadaegh F (2021). Recurrence of a neuroendocrine tumor of adrenal origin: a case report with more than a decade follow-up. BMC Endocr Disord.

[7] Fisher BM, MacPhee GJ, Davies DL, McPherson SG, Brown IL, Goldberg A (1987). A case of watery diarrhoea syndrome due to an adrenal phaeochromocytoma secreting vasoactive intestinal polypeptide with coincidental autoimmune thyroid disease. Acta Endocrinol (Copenh).

[8] Başarslan S, Karakuş A, Çevik M (2014). Unusual a cause of quadriparesis: hypokalemia; a case report. J Exp Clin Med.

[9] Mandreker B, Krishni B, Joel D (2018). A case of acute areflexic, flaccid quadriplegia resulting from acute hypophysitis. Int J Case Rep Images.

[10] Garg SK, Saxena S, Juneja D, Singh O, Kumar M, Mukherji JD (2014). Hyperkalemia: a rare cause of acute flaccid quadriparesis. Indian J Crit Care Med.

[11] Jhamb R, Gupta N, Garg S, Kumar S, Gulati S, Mishra D, Beniwal P (2007). Diffuse lymphomatous infiltration of kidney presenting as renal tubular acidosis and hypokalemic paralysis: case report. Croat Med J.

[12] Wahab A, Panwar RB, Ola V, Alvi S (2011). Acute onset quadriparesis with sine wave: a rare presentation. Am J Emerg Med.

